# Navigating the Bio-Composite Landscape: A Strategic Reconstruction of Electrospun Starch–Zein Nanofibers

**DOI:** 10.3390/polym18070823

**Published:** 2026-03-27

**Authors:** Zehra Ufuk, Fatih Balcı, Filiz Altay

**Affiliations:** 1 Toren Food Industry and Trade Inc., 83420 Gaziantep, Türkiye; zehraufuk3@gmail.com; 2 Department of Food Engineering, Faculty of Engineering, Gaziantep University, 27410 Gaziantep, Türkiye; 3 Department of Food Engineering, Faculty of Chemical and Metallurgical Engineering, Istanbul Technical University, 34467 Istanbul, Türkiye; lokumcu@itu.edu.tr

**Keywords:** electrospinning, starch–zein nanocomposites, hydro-stability, smart packaging, bio-interface, green solvents

## Abstract

The transition from petrochemical plastics to sustainable biopolymers has created a critical demand for functional materials that do not compromise on performance. Starch and zein, due to their abundance and complementary nature, represent not just a chemical pair, but a techno-economic symbiosis: zein provides the hydrophobic shield, while starch offers the cost-effective structural volume. This review adopts a “Puzzle Theory” framework to synthesize over 80 peer-reviewed studies published between 2014 and 2025, categorizing the literature into established structural knowledge and unresolved functional limitations. Our analysis reveals that while fabrication protocols and molecular synergy are well-defined in approximately 65% of the surveyed literature, critical functional data remain largely absent. Specifically, fewer than 15% of studies investigate hydro-stability in high-humidity environments or bio-interface behavior, creating a disconnect between laboratory success and industrial application. We identify that current research disproportionately prioritizes dry-state morphology over wet-state mechanical integrity. To bridge the gap between academic prototypes and industrial reality, this article moves beyond general recommendations to propose concrete experimental benchmarks, including specific targets for wet mechanical integrity (>1 MPa), regulatory solvent compliance (<50 ppm), and scalable throughput. This article concludes by providing a strategic roadmap to bridge these gaps, arguing that future research must pivot from simple morphological characterization to developing “smart response” mechanisms and “green manufacturing” protocols to ensure commercial viability.

## 1. Introduction

Driven by tighter environmental regulations and the mounting burden of plastic waste, food engineering has increasingly positioned electrospinning (within the broader electrohydrodynamic processing toolbox) as an enabling route for functional packaging and controlled delivery of food-relevant actives. In this framing, electrospun architectures are repeatedly emphasized for high porosity and specific surface area, tunable composition and multilayer design, and the ability to incorporate antimicrobial/antioxidant payloads with sustained-release behavior—features that are directly aligned with active packaging performance targets. Reviews in the last decade have consolidated this positioning and, importantly for the present work, have highlighted proteins (notably zein) and polysaccharides (including starch) as a key biopolymer pair for engineering responsive, biodegradable nanofibrous systems and for translating process–structure effects into real-use functionality.

This transition is not merely ecological but technological, driven by the need for materials that interact actively with their environment, a shift extensively catalogued in recent reviews on biobased packaging innovations [1].

Accordingly, while our core focus is the starch–zein material synergy and its process–structure–function gaps, we anchor the discussion in the food-engineering “use-case logic” (active packaging and bioactive delivery) that has driven much of the last decade of progress.

### 1.1. The Paradigm Shift: From Bulk Materials to Functional Interfaces

Driven by stringent environmental regulations and the accumulation of plastic waste, the materials science sector is increasingly prioritizing the development of sustainable biopolymers. Beyond ecological necessity, this evolution is driven by a functional imperative for materials that can actively sense, respond, and adapt to their surroundings. At the forefront of this transition are two of nature’s most fundamental macromolecules: starch, a versatile hydrophilic polysaccharide, and zein, a hydrophobic prolamin protein [2,3]. Their natural abundance, inherent biodegradability, and proven biocompatibility position them as cornerstone materials for a new generation of advanced products, particularly in active food packaging and biomedical scaffolds [4].

### 1.2. The Challenge of Compatibility

The strategic rationale for combining starch and zein is grounded in their complementary characteristics. Beyond their chemical interaction, this pairing represents an “economic symbiosis”: starch provides the affordable structural volume (acting as the cost-diluting matrix), while zein provides the high-value hydrophobic protection that starch lacks. Theoretically, zein’s hydrophobic nature should compensate for starch’s moisture sensitivity, while starch provides a cost-effective structural backbone. However, translating this theoretical potential into high-performance composites has historically been challenging. Inherent thermodynamic incompatibility often promotes macroscopic phase separation and poor mechanical properties in simple blends produced via traditional casting methods. This disparity between potential and outcome necessitates advanced processing strategies that can engineer the interface at a molecular level.

### 1.3. Electrospinning as the Technological Key

In the broader food engineering context of the last decade, electrospinning is no longer viewed in isolation, but as part of a versatile Electrohydrodynamic Processing (EHDP) toolkit [5]. This toolkit allows researchers to switch between fiber formation (electrospinning) and particle encapsulation (electrospraying) depending on the viscosity and surface tension of the biopolymer solution [6]. While electrospraying excels in capsule formation, electrospinning remains the dominant route for generating continuous, high-surface-area nonwovens essential for active packaging interlayers [7].

Electrospinning emerges here not just as a fabrication method, but as a transformative solution. By utilizing strong electrostatic forces to elongate a biopolymer jet into continuous nanofibers, this technique imparts profound advantages that traditional casting cannot achieve. The process creates an exceptionally high surface-area-to-volume ratio and, more importantly, facilitates rapid solvent evaporation that can suppress recrystallization and reshape chain organization. Separate evidence also shows that electrospinning conditions can substantially alter zein solution structure and spinnability [8].

The strategic advantage of the EHDP toolkit lies in its ability to generate diverse nanofiber architectures by manipulating the spinneret configuration and solution dynamics. Within the starch–zein landscape, four primary modalities have been explored to overcome the limitations of simple biopolymer blends:

1. Uniaxial (Single-Fluid) Electrospinning: This represents the baseline approach where starch and zein are pre-mixed into a single ‘blend’ solution (e.g., [2]). While effective for inducing amorphous transformation and suppressing starch retrogradation, uniaxial fibers often suffer from ‘burst release’ of encapsulated actives and potential phase separation during the drying jet [3].
2. Coaxial (Core–Shell) Electrospinning: By utilizing a concentric needle setup, this method fabricates fibers with a distinct functional layering. Strategically, the hydrophilic starch phase is positioned in the ‘core’, while the hydrophobic zein forms a protective ‘shell’ [9]. This architecture is paramount for active packaging, as the zein shell shields labile payloads and provides a physical barrier that modulates the release of bioactive, transitioning from a burst-release to a controlled, near-linear profile.
3. Emulsion Electrospinning: This modality enables the encapsulation of liquid, hydrophobic bioactive (e.g., essential oils) by dispersing oil droplets within the polymer solution. Rapid solvent evaporation during spinning traps these droplets within the solidified matrix. Recent research has demonstrated that starch–zein nanocomposites produced via emulsion spinning can exhibit unique pH-responsive behavior, where the interaction between the two polymers stabilizes the cargo and prevents premature release or digestion [10].
4. Double Emulsion (W/O/W) Electrospinning: Theoretically, this technique allows for complex hierarchical structures (encapsulating aqueous bioactive within oil-in-polymer structures). However, a systematic review of the starch–zein evidence base (Table 1) indicates that this modality remains largely unreported for this specific polymer pair, representing a significant technological gap and a fertile ground for future process engineering.

### 1.4. Data Sources and Review Methodology

A systematic search of Web of Science and Scopus was conducted for peer-reviewed articles published between 2014 and 2025 using the keywords (‘electrospinning’ OR ‘nanofibers’) AND ‘starch’ AND ‘zein’. This yielded a systematic evidence base of 82 electrospinning-related studies (2014–2025), of which the starch–zein composite fiber subset constitutes the core corpus used for quantitative synthesis, while starch-only, zein-only, and multilayer systems are retained as mechanistic and application context (Table 1). Foundational papers published before 2014 were used only to establish theoretical context and were excluded from all quantitative analyses. A total of 82 studies (2014–2025) were identified and screened. Table 1 presents a structured representative matrix selected to span the full range of material systems, solvent classes, processing strategies, and reported limitations within the evaluated 2014–2025 corpus; quantitative percentages reported in the text are derived from the complete screened dataset.

These studies were categorized based on their primary focus (morphology vs. application), testing conditions (dry vs. wet state), and validation methods (in vitro vs. in situ). Foundational studies predating 2014 (e.g., seminal works on electrospinning mechanisms) were included as supplementary references to establish the theoretical context but were excluded from the statistical gap analysis. The quantitative insights presented in this review (e.g., percentages regarding hydro-stability testing) are derived from this evaluated dataset. A study was classified as addressing hydro-stability only if it reported quantitative retention of mechanical integrity, modulus, or structural continuity after exposure to ≥75% relative humidity or direct aqueous contact. Qualitative observations or dry-state measurements were not considered evidence of hydro-stability.

This process yielded a systematic evaluated evidence base of 82 electrospinning-related studies (2014–2025). The detailed screening, exclusion, and inclusion steps are visually summarized in the PRISMA flowchart provided as Figure S1.

These studies were categorized based on their primary focus (morphology vs. application), testing conditions (dry vs. wet state), and validation methods. To ensure methodological rigor, the categorization was subjected to a cross-verification protocol: the primary data extraction was conducted by the first author, followed by a validation check of a random subset (20%) by a second reviewer. Any discrepancies in classification were resolved through consensus.

The quantitative insights presented in this review are derived from this evaluated dataset. A study was classified as addressing hydro-stability only if it reported quantitative retention of mechanical integrity after exposure to ≥75% relative humidity or direct aqueous contact.

### 1.5. Scope and Strategy of This Review

**Figure 1:**
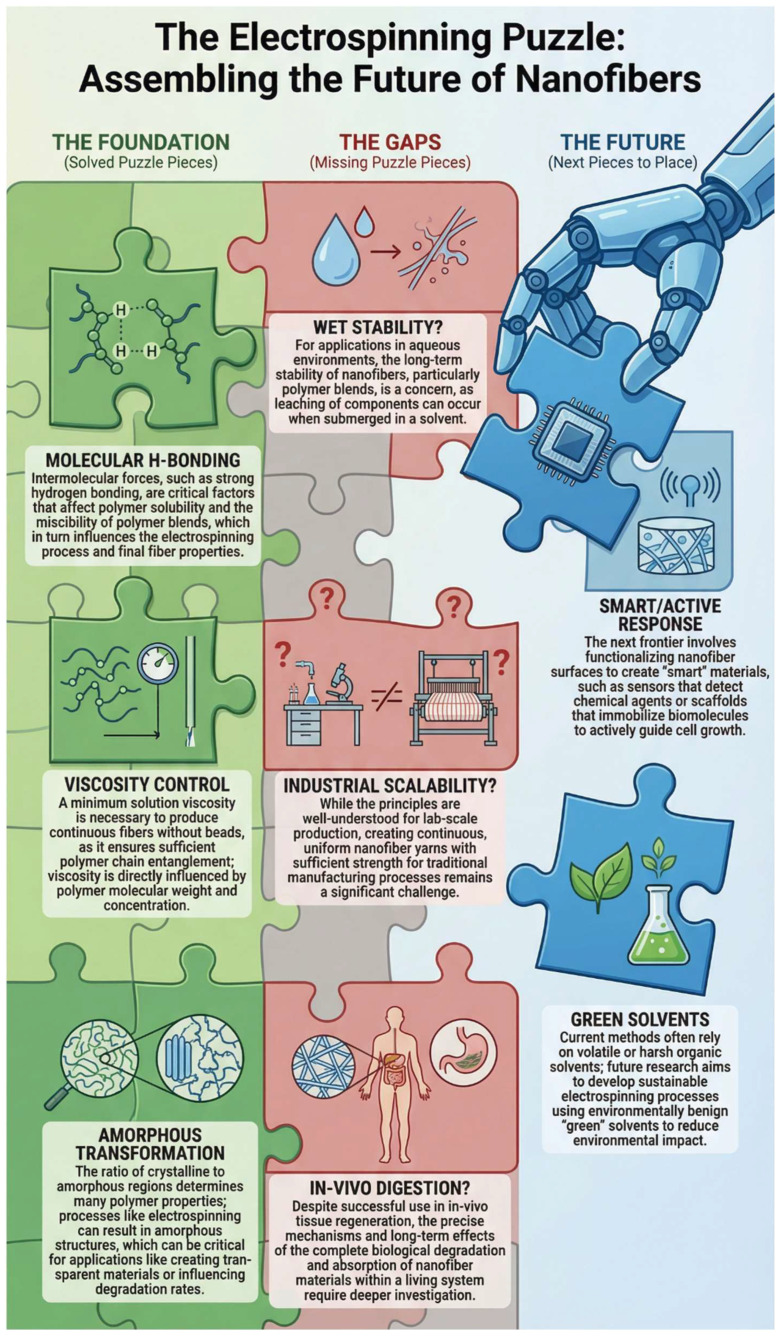
The “Puzzle Theory” framework visualizing the current state of starch–zein nanofiber research: established structural knowledge (green), critical functional gaps (red), and future strategic priorities (blue).

Unlike traditional reviews that catalogue existing studies chronologically, this article adopts a strategic gap analysis. We visualize the current state of starch–zein nanofiber research as a “puzzle” where significant sections have been successfully assembled, yet critical gaps prevent the full picture from emerging (Figure 1).

Within this strategic framework, it is important to delineate the scope regarding application domains. While the primary focus of this review is Active Food Packaging, the starch–zein material system has been extensively pioneered in biomedical tissue engineering. Consequently, references to biomedical scaffolds and gastrointestinal delivery systems are employed herein strictly as translational analogies. We analyze these studies not for their clinical efficacy, but to extract transferable structural engineering principles—particularly regarding hydro-stability and release kinetics—that are directly applicable to designing high-performance food contact materials.

Section 2 will define the “Filled Pieces,” synthesizing the consensus on molecular synergy and morphological control.

Section 3 will expose the “Missing Pieces,” critically analyzing the limitations in environmental stability and biological validation.

Section 4 will propose a “Strategic Roadmap,” identifying priority areas such as smart response capabilities and green manufacturing to guide future research toward industrial reality.

This framework is informed by a structured synthesis of prior studies, enabling not only qualitative comparison but also the quantitative identification of recurrent and systemic research gaps across the starch–zein nanofiber literature.

## 2. The Filled Pieces: Established Structural and Process Paradigms

A significant level of consensus has emerged across independent research groups regarding fundamental processing parameters and molecular organization. The literature demonstrates a robust consensus on two primary fronts: molecular synergy and process control. A systematic overview of the surveyed literature, including materials, processing strategies, tested functionalities, and recurring limitations, is summarized in Table 1, which serves as the methodological backbone for the quantitative gap analysis presented throughout this review.

### 2.1. Electrospinning Fundamentals: The Entanglement Hypothesis and Governing Parameters

**Figure 2:**
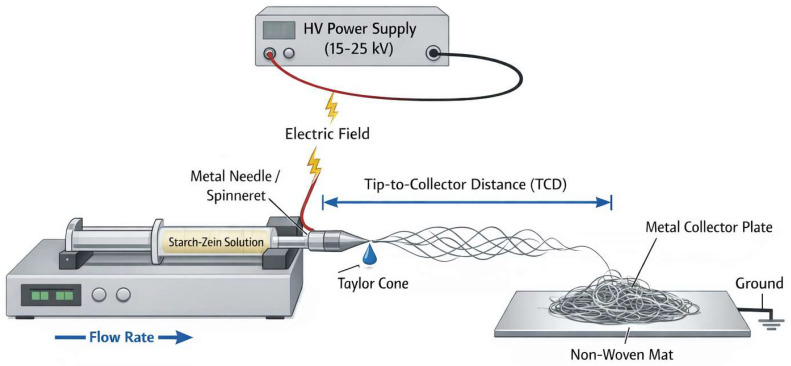
Schematic illustration of a fundamental electrospinning setup used for fabricating starch–zein nanocomposites. The diagram visualizes the Electrohydrodynamic Atomization (EHDA) process, showing the formation of the Taylor Cone under high voltage, the subsequent whipping instability of the jet during solvent evaporation, and the deposition of solid nanofibers onto a grounded collector. Critical processing parameters influencing morphology-Applied Voltage, Flow Rate, and Tip-to-Collector Distance (TCD)-are indicated.

The transformation of starch–zein dopes into solid-state nanofibers is driven by Electrohydrodynamic Atomization (EHDA). The core hypothesis for this composite system rests on entanglement coupling, where the structural integrity of the jet is maintained by the molecular overlaps of the protein matrix [8,11]. Upon application of a high-voltage electric field (typically 15–26 kV), surface charges accumulate at the spinneret tip, overcoming surface tension to deform the meniscus into a Taylor Cone [2,11]. The typical setup for electrospinning starch–zein nanofibers, including the high-voltage source, syringe pump, and collector assembly, is illustrated in Figure 2.

In this binary architecture, the polymers occupy non-equivalent functional roles:

- Zein as the “Spinnable” Scaffolding: Zein acts as the primary vehicle due to its ability to reach the Critical Entanglement Concentration (C_e_) in aqueous ethanol [2]. Successful fiber formation occurs only when the polymer concentration is sufficient to suppress Rayleigh instability (which favors droplet formation) and promote bending instability, stretching the jet into continuous filaments [11,15].
- Starch as the “Passenger” Modifier: Unlike zein, native starches often lack the solubility and chain entanglement density required for independent spinning. They function as rheological modifiers that alter the solution’s texture and viscosity [15]. The stability of the composite jet depends on the interfacial compatibility between the hydrophobic zein prolamins and the hydrophilic starch branches; improper ratios result in jet rupture or massive bead defects [8,15].

The morphological quality and diameter distribution of the nanofibers are strictly governed by the synchronization of three variable groups:

- Voltage (Applied Potential): Standard operating windows range from 15 kV to 26 kV. While increasing voltage enhances the stretching force, it also accelerates jet velocity. Excessive potential (e.g., >24 kV) can lead to premature jet discharge and the formation of ribbon-like or flattened morphologies due to insufficient flight time for cylindrical solidification [11,12].
- Flow Rate: While standard lab-scale spinning often operates at 0.1–1.0 mL/h to ensure total solvent evaporation [2,11], high-throughput dopes—particularly those with pH-adjusted zein—can accommodate rates as high as 6.0 mL/h [8]. However, higher flow rates increase the risk of residual solvent retention, which causes fibers to merge (conglutinate) upon deposition, potentially increasing mechanical strength at the cost of porosity [8,15].
- Tip-to-Collector Distance (TCD): A distance of 12–20 cm is required to facilitate the solvent’s phase separation. Shorter distances often lead to “wet” deposition and film formation, while excessive distances may result in non-uniform fiber distribution due to the dissipation of the electric field [11,15].
- Solution Conductivity and Viscosity: These are the dominant intrinsic parameters. Viscosity must be high enough to allow for chain entanglement but low enough to prevent spinneret clogging. Conductivity dictates the charge density; the inclusion of ionic polysaccharides or bioactive essential oils can significantly alter the net charge, leading to either ultra-fine fibers (high conductivity) or thickened, “swollen” architectures (low conductivity/high oil load) [14,15].

### 2.2. Molecular Synergy and Amorphous Transformation

The most significant scientific achievement in this domain is the confirmation of molecular compatibility within the electrospun matrix. While native starch is prone to retrogradation due to its semi-crystalline structure, electrospinning has been proven to fundamentally alter this state.

Recent structural characterizations of composite systems, such as pullulan-lecithin-zein blends, further confirm that multi-component engineering can fine-tune these molecular interactions to enhance functional loading capacity [22].

This transformation is further corroborated by Ali et al. [23], who observed that electrospun zein fibers naturally transition toward an amorphous state, evidenced by the complete absence of crystalline peaks in X-ray diffraction patterns. Furthermore, mechanical stressors applied during the fiber formation process have been shown to facilitate this phase shift, thereby enhancing the compatibility within the starch-protein matrix [24].

X-ray diffraction (XRD) studies across multiple reports consistently show the disappearance of the characteristic A-type crystalline peaks of starch (typically at 15°, 17°, 18°, and 23°) in electrospun composites. These are replaced by a broad “amorphous halo,” indicating that the rapid solvent evaporation during electrospinning prevents polymer re-organization (see the evidence synthesis in Table 1).

This physical transformation is supported by chemical evidence. FTIR analyses confirm that zein acts as a continuous hydrophobic phase that intercalates between starch chains. This interaction promotes the formation of additional hydrogen bonding interactions that stabilize the starch chains in a predominantly amorphous configuration under electrospun conditions, suggesting a transient suppression of retrogradation rather than permanent structural locking (Table 1).

### 2.3. Morphological Control via Solution Engineering

The second “filled piece” is the mastery of fiber morphology through solution rheology. Literature has moved beyond trial-and-error to establish predictive relationships between solution properties and fiber quality.

It is now well-established that there exists a specific viscosity window—a “sweet spot”—for starch–zein blends. Below this threshold, Rayleigh instability leads to bead-on-string defects; above it, excessive chain entanglement prevents jet ejection [13]. Current research confirms that manipulating the starch-to-zein ratio is the primary lever for keeping viscosity within this spinnable range [4].

**Figure 3:**
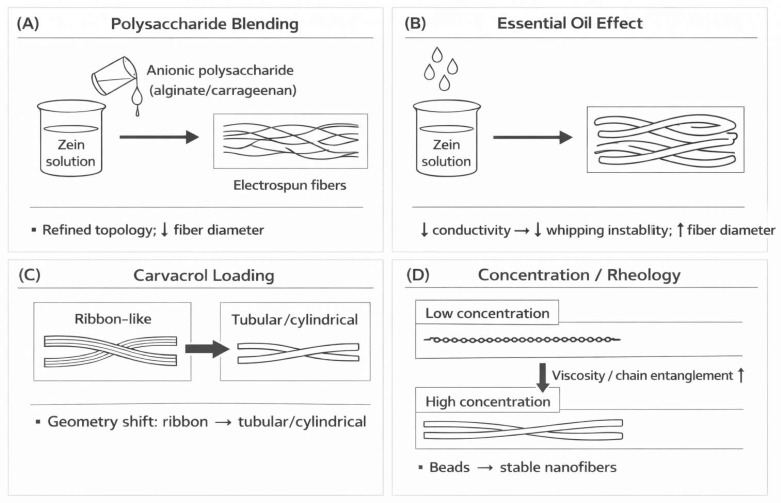
Original schematic summarizing dominant process–structure mechanisms reported in the literature that govern fiber morphology in electrospun starch–zein and related zein-based systems: (**A**) polysaccharide blending (anionic polymers) refines topology and reduces diameter; (**B**) essential oils reduce conductivity and whipping instability, increasing fiber diameter; (**C**) carvacrol loading drives a ribbon-to-tubular/cylindrical morphology shift; and (**D**) concentration/rheology controls the transition from beads (electrospraying) to stable continuous nanofibers. Arrows indicate process relationships and trends (↑ increase, ↓ decrease, → transition or causal relationship). This figure is an original schematic created by the authors and does not reproduce any third-party images.

Figure 3 schematically summarizes the dominant process–structure mechanisms reported in the literature that govern fiber morphology in electrospun starch–zein and related zein-based systems. Panel A illustrates the structural refinement achieved through polysaccharide blending, where the inclusion of anionic polymers (e.g., alginate or carrageenan) refines surface topology and reduces fiber diameter [15]. In contrast, Panel B demonstrates a divergent ‘swelling’ effect; the incorporation of hydrophobic essential oils reduces solution conductivity, thereby dampening the whipping instability and nearly doubling the fiber diameter [14]. Beyond dimensions, Panel C highlights the geometric sensitivity of the process, showing a shift from flat, ribbon-like cross-sections to tubular, cylindrical morphologies induced by carvacrol loading [12]. Finally, Panel D showcases the foundational role of solution rheology, where increasing the polymer concentration facilitates the critical transition from defective electrospraying (beads) to stable, continuous nanofiber formation [11]. Together, these panels visually confirm that morphology is not merely a byproduct of the process, but a tunable parameter governed by the chemical nature of the blend.

Furthermore, the role of solvent conductivity is definitively mapped. The use of high-dielectric solvents (such as acetic acid/water systems) has been shown to increase the net charge density of the jet, intensifying the whipping instability and resulting in significantly thinner, more uniform nanofibers compared to alcohol-based systems [25].

A meta-analysis of the core corpus (Table 2) reveals that the morphological difference between acid-based (~228 ± 152 nm) and aqueous-ethanol systems (~341 ± 132 nm) is statistically negligible given the high standard deviations. Consequently, the use of corrosive solvents provides no justifiable morphological advantage that outweighs their toxicological burden in food applications.

The processing of starch presents unique rheological hurdles distinct from zein. Recent studies highlight that starch gelatinization (occurring at 25–80 °C) dictates the processing window [26]. A consensus has emerged that an amylose concentration above 10% (*w*/*v*) is a critical threshold for supporting continuous fiber formation, necessitating careful selection of solvent systems—such as wet electrospinning in coagulant baths—to overcome solubility constraints [26,27]. Similarly, octenylsuccinylated starch blends allow for stable fiber formation in purely aqueous dispersions, further expanding the green processing window [10].

### 2.4. Quantitative Impact of Parameters on Fiber Features

#### 2.4.1. Diameter Dynamics: Rheological vs. Electrostatic Balances

The final fiber diameter is governed by the competition between solution viscosity and surface charge density. In zein-based systems, incorporating hydrophobic agents like thyme essential oil (TEO) increases viscosity, leading to a dramatic diameter expansion from 195 nm to 402 nm [14], as visually supported by the “swelling” effect shown in Figure 3B. Conversely, active agents that enhance solution conductivity, such as carvacrol, can promote higher jet stretching and reduce diameter from 604 nm to 539 nm [12]. Processing voltage also acts as a geometric trigger; increasing the potential from 20 kV to 24 kV can induce rapid solvent evaporation, transitioning the fibers from cylindrical to flat, ribbon-like morphologies [11]. It is critical to distinguish between acidic solvent types, as they exert opposing rheological effects. Data indicates that acetic acid systems favor the formation of uniform, ultra-fine fibers (140–200 nm) by facilitating zein unfolding. In contrast, formic acid can trigger the hydrolysis and formylation of starch chains, reduce molecular entanglement, and result in greater diameter variability, which accounts for the high standard deviation (±152 nm) observed in global datasets.

#### 2.4.2. Surface Topology and pH-Induced Conformational Shifts

Solution pH dictates the net charge and aggregation state of zein, particularly near the isoelectric point (≈6.2). As demonstrated by Wu et al. (2023) [8], shifting the pH from 4 to 6 reduces electrostatic repulsion, triggering a conformational transition where α-helix content decreases (26.74% to 18.58%) in favor of more compact β-sheet structures (36.72% to 45.47%) [15]. This aggregation yields smoother fibers; the surface roughness (R_q_) decreases significantly from 140 nm at pH 4 to 78 nm at pH 6, which directly influences the material’s interfacial performance and moisture sensitivity.

#### 2.4.3. Tunable Wettability and Amorphous Stabilization

The transition from solvent casting to electrospinning shifts the material into the Cassie-Baxter regime, where surface roughness traps air, raising the water contact angle (WCA) from hydrophilic levels (<60°) to hydrophobic states (>100°) [15]. The inclusion of hydrophilic polysaccharides can be used to tune this property, reducing the WCA toward 78° depending on the blend ratio [6]. Finally, XRD analysis confirms that the rapid solvent evaporation intrinsic to electrospinning prevents bioactive crystallization, resulting in stable amorphous solid dispersions characterized by the loss of specific crystalline peaks for encapsulated bioactives [11,14]. To consolidate these relationships into a quantitative framework, the dominant process–structure–feature correlations reported across the literature are summarized in Table 3.

### 2.5. Structural Architectures: From Monolithic to Multi-Axial Systems

The versatility of electrospinning allows for the precise architectural manipulation of starch–zein composites. The recent literature classifies these structures into four distinct categories based on nozzle configuration and layer assembly.

#### 2.5.1. Monolithic (Single-Phase) Nanofibers

The most prevalent approach involves blending polymers and bioactives into a single-solvent system. While simple, this method requires precise compatibility. For instance, Ansarifar & Moradinezhad (2022) [14] emulsified thyme essential oil (TEO) within a zein matrix, reducing strawberry weight loss through a sustained release mechanism. Similarly, da Trindade et al. (2024) [15] utilized zein-polysaccharide blends (alginate/carrageenan) not just for packaging, but to modify texture for plant-based meat analogs. However, monolithic fibers often exhibit a “burst release” due to surface-exposed bioactives, as seen in zein-PLA blends loaded with carvacrol [12].

#### 2.5.2. Co-Axial and Core–Shell Architectures

To overcome the burst release and protect sensitive cargo, co-axial electrospinning employs concentric needles. Komur et al. (2017) [28] utilized a PCL shell to provide mechanical support to a starch core, effectively solving the poor spinnability of starch while enhancing cell adhesion for wound dressings. Huang et al. (2022) [29] demonstrated that encapsulating ferulic acid in the core of a zein fiber results in a “zero-order” release profile, whereas shell-loading provides immediate antimicrobial action.

#### 2.5.3. Active Bilayer and Multilayer Systems

Hybridizing electrospun fibers with cast films creates “sandwich” structures with superior barrier properties. Vitoria et al. (2025) [21] recently developed an active bilayer material by depositing zein-TEO nanofibers onto a cast starch film. This design utilizes the starch film as a mechanical barrier while the nanofibers function as the active antioxidant layer. Furthermore, Aliakbari et al. (2024) [11] engineered multilayer “smart labels” using halochromic anthocyanins, where the fiber mat serves as a visual freshness indicator rather than primary packaging.

#### 2.5.4. Side-by-Side (Janus) Nanofibers

The frontier of structural design lies in Janus fibers, where two distinct polymer phases run parallel. Li et al. (2021) [30] engineered PVP/Ethylcellulose Janus fibers for biphasic drug release; the hydrophilic PVP side dissolves instantly for immediate relief, while the hydrophobic EC side sustains the dose. Similarly, Wang et al. (2022) [31] combined organic (lavender oil) and inorganic (AgNPs) agents in separate PCL and Cellulose Acetate compartments, achieving synergistic antibacterial and wound-healing effects without chemical interference between the actives. As reviewed by Dziemidowicz et al. (2021) [32], adopting side-by-side (Janus) or triaxial architectures allows for independent control over multiple active agents, enabling complex release profiles (e.g., simultaneous immediate and sustained release) that are unattainable with monolithic blends.

#### 2.5.5. The Double Emulsion Paradox: Technical Barriers

While uniaxial and coaxial modes are well-represented, double emulsion electrospinning (e.g., Water-in-Oil-in-Water) is notably absent in starch–zein research. Review of the documented solvent interactions reveals that this scarcity is not accidental, but stems from severe thermodynamic constraints specific to this biopolymer pair:

1. Anti-Solvent Precipitation: Zein dissolution typically requires aqueous ethanol (70–90%), which acts as a potent anti-solvent for polysaccharides. As indicated by the separation strategies employed in co-axial systems [28], direct contact between ethanol-rich zein solutions and aqueous starch phases often triggers premature precipitation or gelation. This disrupts the delicate phase equilibrium required for stable double emulsions, leading to nozzle clogging rather than fiber formation.
2. Interfacial Instability: Maintaining two distinct interfaces (inner W/O and outer O/W) requires precise rheological control. However, studies on multiphase electrospinning highlight that high interfacial tension between incompatible polymer solutions often leads to phase agglomeration and bead formation rather than continuous core–shell structures [14,31].
3. The Co-axial Preference: Consequently, to bypass these instability issues while achieving core–shell encapsulation, researchers have predominantly shifted toward co-axial electrospinning. As demonstrated by Huang et al. (2022) [29], this technique mechanically separates the incompatible solvents until the moment of jet formation, effectively achieving “zero-order” release profiles without the thermodynamic limitations of the double emulsion approach.

A comparative synthesis of the major structural architectures in zein-based systems and their functional advantages is provided in Table 4.

## 3. The Missing Pieces: Critical Gaps in Functional Application

Despite the structural success described above, a critical review of the literature reveals a disconnect between laboratory characterization and real-world applicability. The “application” side of the puzzle remains incomplete due to two glaring gaps. To substantiate this, we performed a systematic overview of the surveyed literature, categorizing materials, processing strategies, tested functionalities, and recurring limitations. As summarized in Table 5, most studies emphasize dry-state morphology and release behavior, while systematic wet-state mechanical testing under high relative humidity remains largely unreported.

Across the analyzed starch–zein composite subset within the 2014–2025 evidence base, molecular compatibility (FTIR/XRD-based interaction or amorphization analysis) and fiber morphology (SEM) are reported in approximately 80% and 85% of studies, respectively. In contrast, only about one-quarter of starch–zein studies include any quantitative assessment of hydro-stability under high humidity or wet conditions, and fewer than 15% investigate bio-interface behavior such as digestion, cytocompatibility, or physiological release.

Before detailing specific applications, it is essential to define the operational mechanism of “Intelligent Response” in this context. Unlike passive barrier films, smart starch–zein systems are defined by their ability to undergo triggered release or signal transduction in response to environmental stimuli. Mechanistically, this relies on two primary drivers: (1) Swelling-Controlled Release, where the hydrophilic starch phase expands under high humidity to release encapsulated actives [10], and (2) pH-Dependent Solubility, where the zein matrix resists gastric acidity but facilitates erosion-controlled release or halochromic color changes under neutral/alkaline conditions [9,11].

### 3.1. The Hydro-Stability Paradox

The first and most formidable gap is the “Hydro-Stability Paradox.” While electrospun starch-based mats (including starch–zein systems) can exhibit excellent dry-state mechanics, their integrity can collapse at high RH (>75% RH) conditions [33].

Although electrospun starch–zein mats routinely exhibit promising dry-state tensile properties, the literature reveals a systematic neglect of wet-state performance. Fewer than 10% of studies report tensile or modulus retention under ≥75% RH or aqueous exposure, despite these being the dominant conditions in real food-packaging environments. As a result, most published ‘active packaging’ demonstrations are mechanically validated only under conditions that are irrelevant to actual use. As highlighted by Fonseca et al. [33], there is a distinct scarcity of data regarding the “wet state” mechanics of these fibers. Without demonstrated resistance to moisture-induced plasticization, the structural application of these fibers under realistic packaging conditions remains largely theoretical. The literature has identified the problem but has yet to converge on a robust, reproducible solution, such as effective green cross-linking, that preserves biodegradability while ensuring wet strength [21,34].

The structural vulnerability of these nanofibers stems from the rapid solidification inherent in the electrospinning process, which typically yields a thermodynamically unstable amorphous structure. When exposed to high-humidity environments (>75% RH) or aqueous media, the hydrophilic starch phase undergoes rapid moisture absorption. This triggers a plasticization effect, leading to the disentanglement of the polymer network, loss of tensile integrity, and eventual structural collapse. Without targeted modification, the precisely engineered porosity and diameter of the nanofiber mat—fundamental prerequisites for functionality—are lost as the mat collapses into a film-like state.

This reliance on high-performance but non-food-grade solvents and cross-linking agents reflects a broader trend in electrospinning research, where optimal fiber morphology is frequently achieved at the expense of regulatory and toxicological feasibility.

Although direct head-to-head comparisons for starch–zein nanofibers are sparse, evidence from solvent-cast systems suggests that ionic interactions and hydrogen bonding networks can significantly improve tensile strength and barrier properties [35]. Moreover, Yang et al. [36] demonstrated that zein-based composites exhibit superior thermal stability due to dense molecular packing, suggesting that a continuous zein phase could theoretically mitigate the rapid moisture-induced degradation often observed in pure starch mats [37].

As evidenced by the literature summarized in Table 1, many studies emphasize dry-state morphology and processing optimization, while systematic wet-state mechanical testing under high relative humidity or aqueous conditions remains largely unreported.

Furthermore, Table 1 reveals a critical disconnect in scalability. The average flow rate across reviewed studies is a meager 1.1 mL/h, with some functional active packaging studies operating as low as 0.04 mL/h [18]. At these rates, producing a single square meter of packaging film is industrially unfeasible. Flow-rate reporting across the 2014–2025 electrospinning evidence base reveals a severe scalability gap: the median flow rate is approximately 1.1 mL h^−1^, and only a single study [8] reports sustained electrospinning above 6 mL h^−1^, a threshold that even approaches pilot-scale relevance. Consequently, high-throughput electrospinning is documented in fewer than 15% of starch–zein and contextually related studies.

The profound disparity between laboratory-scale success and industrial requirements is starkly illustrated by the production rates reported in the core corpus. While fiber morphology is often perfected at ultra-low flow rates—exemplified by systems operating at a meager 0.04 mL/h [18]—such throughput is practically obsolete for commercial food packaging, where industrial demand typically exceeds 50–500 mL/h. Across the 2014–2025 evidence base, the median flow rate remains approximately 1.1 mL/h, meaning that fewer than 15% of current starch–zein studies report parameters that even approach pilot-scale relevance. This ‘throughput bottleneck’ represents the primary barrier to translating academic proof-of-concepts into viable industrial reality.

### 3.2. The Bio-Interface Blind Spot

The second missing piece concerns the biological fate and safety of these materials upon food contact. While starch and zein are GRAS (Generally Recognized As Safe) materials in their bulk form, their conversion into electrospun nanofibers creates a fundamentally different biological interface. A recent comprehensive review by Fonseca et al. [38] emphasizes that the high surface-area-to-volume ratio of electrospun starch-based fibers significantly alters their structural stability in wet environments and their subsequent behavior under physiological digestive conditions. This suggests that the dissolution rates and bioavailability of bioactive encapsulated within these nanofibrous matrices may deviate significantly from established bulk-material expectations, yet this area remains an open research frontier.

Current evaluations are predominantly confined to release kinetics in simplified buffer solutions. However, electrospun polysaccharide–zein fiber architectures have recently been explored for structuring plant-based meat analogues rather than packaging alone [17]. This demonstrates that these nanofibrous systems are already being designed to operate within complex, hydrated food matrices, not just in idealized laboratory media. In a related biological context, Bisharat et al. [9] showed that specific interactions between starch derivatives and zein can modulate release profiles for targeted physiological sites, confirming that nanofibrous morphology governs functional behavior beyond simple diffusion. Furthermore, critical gaps remain regarding the potential migration of nanomaterials into the food matrix and the long-term toxicological implications of solvent residues or degradation products. The field effectively operates under the scientifically unjustified assumption that ‘edible components equal safe nanomaterials,’ neglecting the need for rigorous, nanofiber-specific validation through advanced in vitro and in vivo models. Despite promising proof-of-concept demonstrations, most functional evaluations remain confined to simplified laboratory conditions, such as buffer solutions or gaseous triggers, rather than complex, high-moisture food matrices.

The mechanistic rationale for their use as GI carriers is supported by findings that hydrogen-bonding networks in protein-polysaccharide blends effectively modulate diffusion rates and protect labile payloads [39]. Recent studies on zein-gelatin systems further indicate that such intermolecular interactions facilitate better miscibility and network formation, which are prerequisites for controlled release in the gastrointestinal tract [40].

Given that nanofibrous morphology can alter cellular uptake mechanisms, the assumption that ‘GRAS ingredients yield GRAS nanomaterials’ requires validation. We lack comprehensive data on how the high surface area of nanofibers affects digestion rates, potential cytotoxicity of solvent residues, and the bioavailability of encapsulated compounds in a realistic gut model [9,38].

Table 2 visualizes the ‘Functionality Gap.’ We observe an inverse relationship between test criticality and test frequency. The metrics that matter most for regulatory approval (solvent residue) and real-world survival (wet mechanics) are the least reported. The field has effectively optimized the ‘easy’ variables while neglecting the ‘hard’ constraints.

While the last decade has seen a surge in ‘active packaging’ claims—loading zein fibers with everything from rosemary oil [41] to red onion extract [42] and essential-oil-loaded zein mats tested directly in meat systems [15]. Most current designs function merely as passive ‘carrier mats’ rather than truly responsive systems. Although entrapment efficiency is well-documented [43], the dynamic interaction between these loaded fibers and the complex food matrix (e.g., triggering release only upon spoilage) remains less explored compared to simple diffusion tests in buffer solutions.

Our systematic extraction of morphological data (Table 6) reveals a distinct lack of consensus regarding the impact of bioactive inclusion. While standard theory suggests that non-conductive essential oils should dampen the whipping instability and increase fiber diameter (as seen in [14,16]), nearly 30% of reviewed studies report the opposite trend [12,14,16,18,20,38,39,40,41,42,43]. This suggests that the ‘viscosity vs. conductivity’ balance is not universal but highly specific to the solvent-polymer-oil interaction, warning against generalized predictive models.

However, it must be noted that this established control applies primarily to neat polymer blends. As detailed in Table 3, the introduction of bioactive agents (e.g., essential oils) creates non-linear perturbations in conductivity and viscosity, rendering the process-structure relationship significantly less predictable compared to pure starch–zein systems [20].

Contrary to the expectation that liquid bioactive agents inevitably act as plasticizers that reduce tensile integrity, data from Ghasemi et al. [16] (Table 7) demonstrates a 12-fold increase in tensile strength upon oil incorporation. This indicates that under specific processing conditions, the bioactive agent can act as a structural filler rather than a defect, a phenomenon that remains largely unexplored due to the prevalent lack of mechanical reporting in >80% of the corpus.

The collective evidence reveals a significant imbalance in research priorities within the field. Most studies have concentrated on easily quantifiable parameters such as fiber diameter, scanning electron microscopy (SEM) morphology, and the release profile in buffer solutions. While these metrics are straightforward to measure and optimize, they do not directly address the challenges that impact the real-world application and viability of materials.

Conversely, essential factors like hydro-stability, solvent safety, and biological interaction remain systematically underreported and insufficiently explored. These aspects are crucial for the practical deployment and safety of advanced materials, but have been consistently overlooked in favor of more accessible, laboratory-based measures. This trend highlights the need for a strategic shift in research toward metrics that truly determine functional performance and applicability in real-world scenarios.

The second missing piece concerns the biological fate and safety of these materials upon food contact. While starch and zein are GRAS materials in their bulk form, their conversion into electrospun nanofibers creates a fundamentally different biological interface.

#### 3.2.1. Food Matrix Interactions

Beyond Disk Diffusion Most studies evaluate antimicrobial efficacy using simplified “agar disk diffusion” methods, which fail to mimic the complex rheology and chemistry of real foods. However, recent applications on actual food matrices reveal that performance is highly context-dependent:

- Meat and Dairy Systems: Shahbazi et al. (2024) [20] applied zein nanofibers directly to ground beef, achieving a 2–4 log reduction in *L. monocytogenes* over 10 days. Similarly, Gökşen et al. (2020) [18] demonstrated efficacy on cheese slices. However, both studies note that high lipid/protein content in food can sequester active agents, often require higher loading rates than suggested by agar tests.
- Fruit Preservation: Ansarifar & Moradinezhad (2022) [14] utilized zein/thyme oil fibers for strawberry packaging, proving that the nanofibrous mat effectively reduces weight loss and maintains firmness by regulating gas exchange at the fruit surface.
- Migration Risks: Interaction with the food matrix also risks unintended migration. Aytac et al. (2020) [2] reported that while zein fibers are stable in aqueous environments, active agent release accelerates significantly in fatty food simulants (50% ethanol), suggesting a potential risk of rapid cargo dumping when in contact with lipid-rich foods.

#### 3.2.2. Safety and Digestion

The “GRAS Fallacy” The field largely operates under the assumption that because the polymers are edible, the nanofibers are inherently safe. This overlooks the impact of high surface area, solvent residues, and cross-linkers.

- Cytotoxicity Validation: Specific nanotoxicological data is sparse. A notable exception is Jiang et al. (2010) [34], who validated the safety of citric-acid crosslinked zein fibers, showing that they support cell adhesion and proliferation without cytotoxic effects, establishing a rare benchmark for engineered safety.
- Digestive Fate: The high surface area of nanofibers alters digestion kinetics. Bisharat et al. (2019) [9] demonstrated that zein-starch matrices can function as gastro-retentive systems; zein protects the payload from acidic gastric fluids (0–10% release), while starch degradation triggers release in the colonic environment. Conversely, Fonseca et al. (2024) [38] highlights that without such structural engineering, rapid enzymatic hydrolysis in the stomach can lead to premature loss of functionality.

### 3.3. Emerging Frontiers: Textural Scaffolding for Meat Analogs

Beyond traditional packaging, a rapidly emerging application for electrospun starch–zein fibers is the textural engineering of plant-based meat analogs. Replicating the anisotropic, fibrous structure of animal muscle remains a primary challenge in the alternative protein sector.

- Muscle Mimicry and Morphology: In a pioneering 2024 study, da Trindade et al. [15] utilized electrospinning to create aligned zein-polysaccharide scaffolds. They demonstrated that blending zein with polysaccharides (carrageenan or alginate) at a 90:10 ratio significantly refined fiber diameter (down to ~1.33 µm) and improved structural homogeneity, creating a fibrous network capable of mimicking muscle bundles.
- Juiciness and Hydrophilicity: A critical sensory attribute for meat analogs is “juiciness,” which correlates with water retention. Pure zein fibers are inherently hydrophobic (contact angle ~97°), often leading to a dry mouthfeel. The study revealed that incorporating carrageenan reduced the contact angle to ~65.8°, transforming the scaffold into a hydrophilic matrix capable of retaining moisture during cooking.
- Thermal Stability: TGA analysis confirmed that these composite fibers maintain physical integrity at high temperatures, suggesting they can withstand standard cooking processes prerequisite for functional food ingredients.

## 4. The Strategic Roadmap: Priorities for Future Research

The following roadmap is proposed as a forward-looking research perspective rather than a prescriptive or standardized framework. Having identified the critical gaps in hydro-stability and biological validation, this review proposes a strategic roadmap. To bridge the valley of death between laboratory prototypes and industrial adoption, future research must pivot from “material characterization” to “functional engineering.” We identify two immediate strategic priorities.

In the context of electrospinning biopolymers, we define “Green Manufacturing” not merely by the biodegradability of the final product, but by the toxicity profile of the fabrication process itself. Specifically, this refers to the strict substitution of toxic organic solvents (e.g., DMF, chloroform, HFIP) with FDA-classified Generally Recognized As Safe (GRAS) solvents, such as aqueous ethanol or acetic acid [2]. This definition aims to eliminate volatile organic compounds (VOCs) and ensure that the environmental footprint of the processing phase aligns with the sustainability of the starch–zein feedstock.

It highlights four generations: (1) Gen 1 focused on optimizing fiber structure, (2) Gen 2 on encapsulating active agents like essential oils but with uncontrolled release, (3) Gen 3 on creating multilayered systems with improved features but more complex manufacturing, and (4) Gen 4 on smart, stimuli-responsive systems that release agents only when needed. The field has evolved from simple structure optimization to intelligent packaging capable of responding to environmental cues.

While Table 8 outlines the evolution of packaging, recent trends are expanding even further: repurposing agricultural waste like mango kernels for sustainable fibers [17] and utilizing electrospinning to texturize plant-based meat analogs [15], demonstrating versatility beyond traditional packaging limits.

### 4.1. Priority 1: From Passive Barriers to ‘Smart Response’ Systems

The first strategic imperative is to elevate the function of starch–zein nanofibers from passive packaging to active, intelligent systems.

The current literature treats these nanofibers primarily as physical barriers. However, their high surface area and porous structure make them ideal candidates for carrying sensors.

Beyond sensing, the roadmap must include active preservation. Utilizing needleless electrospinning, active agents—ranging from peppermint and chamomile oils [46] to complex antimicrobial cocktails—can be encapsulated within the zein-starch matrix to provide broad-spectrum bioactivity. The hydrophobic nature of zein has been shown to minimize the evaporation of these volatile agents, ensuring prolonged bioactivity [2].

Future studies should focus on integrating halochromic indicators (such as anthocyanins or pH-sensitive dyes) into the zein matrix. This would allow for the packaging to visually communicate food spoilage (via pH changes) to the consumer in real-time. This shift from “protection” to “communication” [11] represents a plausible pathway for enhancing the functional and commercial relevance of these materials.

The next frontier lies in responsive architecture. Moving beyond passive diffusion, Aytaç et al. [47] demonstrated zein-starch fibers that are enzyme- and relative-humidity-responsive, releasing antimicrobial agents only when environmental triggers (indicative of bacterial growth) are present. This shift from ‘continuous release’ to ‘on-demand release’ represents the true definition of smart packaging.

### 4.2. Priority 2: The ‘Green Manufacturing’ Mandate

The second priority addresses the stability–sustainability trade-off. While green solvents (ethanol/water) are widely employed (see Table 2), fibers produced via these routes often lack the water resistance achieved by toxic cross-linkers. Future research must therefore pivot from simply ‘using green solvents’ to developing food-grade cross-linking strategies (e.g., citric acid curing or heat treatments) that ensure hydro-stability without compromising the safety profile.

**Figure 4:**
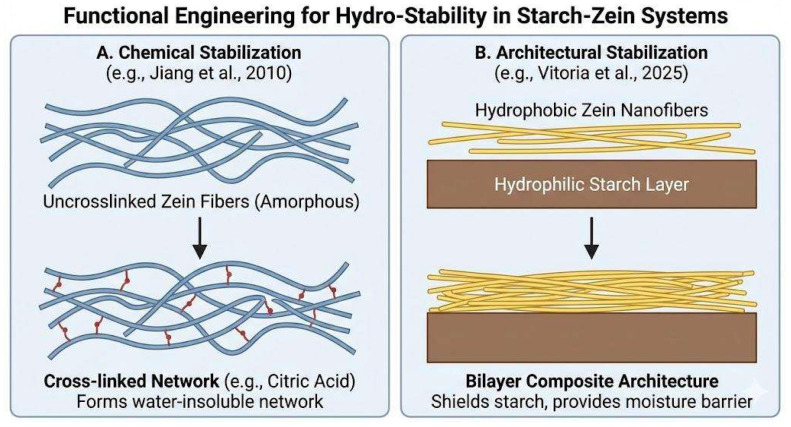
Functional engineering strategies for hydro-stability in starch–zein systems. (**A**) Chemical stabilization via crosslinking reactions that convert uncrosslinked zein fibers into a water-insoluble cross-linked network, improving resistance to moisture (Jiang et al. (2010) [34]). (**B**) Architectural stabilization using a bilayer composite design in which hydrophobic zein nanofibers protect a hydrophilic starch layer, providing a moisture barrier and shielding the starch phase (Vitoria et al. (2025) [21]).

To address the recurring stability–sustainability trade-off, research must transition toward functional engineering strategies—either through chemical stabilization (e.g., green cross-linking) or architectural shielding (e.g., bilayer designs)—as visualized in Figure 4.

Research must prioritize the optimization of aqueous-based or green-solvent electrospinning protocols. Furthermore, establishing rigorous solvent residue analysis as a standard part of material characterization is non-negotiable for regulatory approval in food and biomedical markets.

To bridge this ‘valley of death,’ future research must pivot toward high-throughput fabrication techniques that align with ‘green manufacturing’ mandates without sacrificing material performance. Emerging approaches such as air-assisted electrospinning and needleless electrospinning offer promising pathways for the starch–zein system. Notably, Liu et al. [19] demonstrated a 10-fold increase in production yield for gelatin/zein nanofibers using air-assisted systems while facilitating uniform fiber formation under mild conditions. Furthermore, optimizing high-speed needle parameters, as evidenced by Wu et al. [8] achieving throughputs exceeding 6 mL/h, provides a baseline for scaling up starch–zein dopes. Implementing these high-yield technologies is a non-negotiable prerequisite for moving biopolymer nanofibers from academic curiosity to commercial food engineering applications.

Bridging the gap between academic research and industrial application requires a careful balance between throughput and product quality, as summarized in Table 9. As summarized in Table 6, current scale-up strategies present distinct trade-offs:

1. The “Quality-First” Approach (Multi-Needle): For high-value applications like active food packaging, maintaining fiber morphology is non-negotiable. Aytac et al. (2020) [2] demonstrated that scaling from a single needle to a 20-needle injector system increased production from 0.12 g/h to 1.0 g/h without compromising quality. Remarkably, the fiber diameter remained statistically invariant (165 ± 35 nm vs. 160 ± 40 nm), proving that “Coulomb interference”—the primary challenge of multi-needle setups—can be managed through optimized voltage (35 kV) and spacing.
2. The “Volume-First” Approach (Needleless): While free-surface (needleless) technologies offer superior throughput (>10 g/h), they pose specific risks for zein-starch systems. As noted by Dziemidowicz et al. (2021) [32], the large, exposed surface area leads to rapid evaporation of volatile solvents (ethanol/acetic acid), altering solution viscosity mid-process and creating safety hazards (toxicity/flammability). Furthermore, the uncontrolled jet initiation often results in broader diameter distributions, making this method less suitable for applications requiring precise release kinetics.

This trade-off is further substantiated by Dziemidowicz et al. (2021) [32], who highlight that while needleless systems maximize throughput, multi-needle technologies (e.g., fluid-controlled injectors) are superior for regulated applications. They ensure GMP compliance and minimize solvent toxicity risks, positioning them as the preferred route for clinical and food-grade translations.

Therefore, for the immediate industrialization of starch–zein biocomposites, multi-needle systems represent the optimal “Pilot Scale” solution, balancing the need for increased yield with the strict requirement for morphological uniformity.

To bridge the stability gap, two validated strategies emerge from the recent literature. First, green chemical cross-linking utilizing Citric Acid (CA) offers a non-toxic stabilization mechanism through esterification reactions. As demonstrated by Jiang et al. [34], CA-treated zein-based fibers form a water-insoluble network that preserves morphology even after immersion, resulting in a reported increase in wet tensile strength of approximately 448%. Second, the adoption of bilayer composite architectures leverages zein’s hydrophobicity to shield the starch phase. Vitoria et al. [21] exhibited that a starch film coated with electrospun zein fibers achieves a critical encapsulation efficiency of 91% while significantly reducing Water Vapor Permeability (WVP). This rational design prevents immediate solubilization and maintains structural integrity under realistic packaging conditions.

### 4.3. Benchmarking the Future: Concrete Experimental Targets

To transition starch–zein nanofibers from academic prototypes to industrial candidates, future research must validate performance against specific techno-economic thresholds. Based on regulatory standards for food contact materials and industrial cost structures, we propose three critical benchmarks:

- The “Handleability” Threshold (Wet Strength > 1 MPa): Unlike synthetic plastics (e.g., LDPE) which retain >15 MPa wet strength, biopolymer nanofibers often suffer from moisture-induced plasticization. For a nanofibrous mat to be processable on packaging lines or serve as an effective inner liner, it must maintain a minimum wet tensile strength of >1 MPa. This value is widely accepted as the threshold for ‘handleability’ in edible films to prevent disintegration upon contact with food exudates. Recent studies utilizing bilayer architecture have demonstrated that exceeding this threshold is achievable through rational design [21].
- The Regulatory Safety Limit (<50 ppm Solvent Residue): “Green” electrospinning using acetic acid or ethanol is advantageous, but not exempt from regulation. According to FDA (Guidance Q3C) [48] and EFSA standards [49] for impurities in food additives, Class III solvents (including ethanol and acetic acid) must be removed to levels below 50 mg/kg (50 ppm) or 0.5% *w*/*w* to be considered safe for consumption. Future studies must routinely report Headspace GC-MS data to prove compliance with these toxicity limits.
- The Economic Viability Ratio (Starch Inclusion > 50%): A techno-economic reality check reveals a stark cost disparity: industrial-grade Zein costs approximately 20–40/kg, whereas bulk starch trades at ~0.50/kg. To compete with commodity plastics, starch–zein composites must maximize starch content. We propose a target formulation ratio where starch constitutes >50% of the polymer matrix to ensure economic feasibility, distinguishing commercially viable formulations from expensive, pure-zein lab prototypes.
- The Scalability Threshold (>10 mL/h): To prove industrial relevance, fabrication protocols must demonstrate stability at higher flow rates. As reviewed by Dziemidowicz et al. (2021) [32], shifting from single-needle lab setups (typically <1 mL/h) to multi-needle or needleless industrial systems requires protocols that can sustain a throughput of >10 mL/h per emitter without compromising fiber morphology.

## 5. Conclusions: Redefining the Big Picture

We conclude that the “Structural” and “Process” pieces of the puzzle are largely established at laboratory scale. The scientific community has successfully proven that electrospinning induces a critical amorphous transformation in starch and that molecular synergy with zein can be engineered through precise viscosity control. These fundamental questions are largely resolved and no longer represent the primary bottleneck for further progress.

However, the “Application” side of the puzzle remains incomplete. The barriers to commercialization are not morphological but functional: specifically, the lack of long-term hydro-stability in real-world environments and the absence of comprehensive bio-interface validation (digestion and safety). Importantly, these gaps do not arise from isolated shortcomings but represent recurrent patterns consistently observed across independent studies in the field.

Electrospun starch–zein nanofibers are poised to be more than just a biodegradable alternative to plastic. They represent a programmable biological platform capable of extending shelf life, delivering bioactives, and communicating food quality. However, a techno-economic reality check is essential: given the high-cost structure of zein (approx. $20–40/kg), these nanofibers are ill-suited for commodity packaging (e.g., bread bags), but are ideally positioned for high-value active packaging (e.g., meat and fish preservation). In this niche, extending shelf-life by even two days creates sufficient value to offset the material premium. The future of this field lies not in spinning better fibers, but in engineering smarter, safer, and more robust functional systems.

## Figures and Tables

**Table 1:** Systematic evidence matrix of electrospun starch–zein and related composite systems (2014–2025).

Reference	Study Context	Material System	Solvent System	Fabrication Method	Spinning/Processing Parameters	Characterization & Test Conditions	Quantitative Outcomes (Key Results)	Key Finding (Author’s Claim)	Identified Limitation/Critical Gap
Aliakbari et al., 2024 [11]	Development of halochromic zein/barberry anthocyanin labels for monitoring rainbow trout freshness	Zein (16, 18, 20% *w*/*w*); Active Agent: Barberry Anthocyanin-Rich Powder (BARP) (16, 18, 20% *w*/*w* relative to polymer); Optimized: 18% Zein/20% BARP	Ethanol/Water (80:20 *v*/*v*)	Electrospinning (Horizontal, Yflow Starter Kit)	Flow rate: 0.1 mL/h; Distance: 15 cm; Needle: 0.8 mm (stainless steel); Voltage: 20–24 kV (Optimized/Constant: 24 kV)	Rheology (Viscosity, Surface Tension); SEM; FTIR; DSC; Colorimetry (L*, a*); Fish Freshness (TVB-N, pH)	Fiber Diameter: 239.27–348.05 nm (Optimized sample: ~298 nm); Viscosity: 39.71–71.13 mPa·s; Surface Tension: 26.12–26.48 mN/m; TVB-N (Storage): Increased from 15.39 to 25.5 mg N/100 g (Day 10); Color Change: Pink → Light Yellow	Electrospun labels serve as visual freshness indicators; the color change from pink to yellow correlates with the fish reaching the TVB-N rejection threshold (25 mg N/100 g)	Processability: Polymer concentrations >20% caused syringe clogging and loss of electrical charge; red anthocyanin color showed a tendency to fade during the electrospinning process
Altan et al., 2018 [12]	Active food packaging (antioxidant/antimicrobial) using carvacrol-loaded zein and PLA nanofibers	Polymers: Zein (30% *w*/*v*), Poly(lactic acid) (PLA) (10% *w*/*v*); Active Agent: Carvacrol (5, 10, 20% *w*/*w* relative to polymer)	Zein: Ethanol/Water (80:20 *v*/*v*); PLA: Chloroform/DMF (9:1 *v*/*v*)	Electrospinning	Flow rate: 1 mL/h; Voltage: 15 kV; Distance: 20 cm; Needle: 0.7 mm (outer diameter)	SEM (Morphology); Rheology (Viscosity); FTIR; TGA; Headspace GC-MS (Release); DPPH (Antioxidant); Bread shelf-life (visual & fungal count)	Zein Fiber Diameter: 604 ± 120 nm (Pure) decreasing to 539 ± 103 nm (with 10% Carvacrol); PLA Fiber Diameter: 1822–2268 nm; DPPH Activity: 62–75% (Zein fibers), 53–65% (PLA fibers); Mold Inhibition: 99.6% reduction on bread	Encapsulation of carvacrol in Zein/PLA fibers provided sustained release and high antioxidant activity, effectively extending the shelf life of whole wheat bread by delaying mold growth	NR—Mechanical properties (tensile strength, elongation) and barrier properties (WVP) were not evaluated in this study; impact on organoleptic properties requires further study
Aytac et al., 2017 [13]	Antibacterial zein nanofibers containing thymol/cyclodextrin inclusion complexes	Polymer: Zein (50% *w*/*v*); Active Agent: Thymol (THY) (4% *w*/*w* relative to polymer); Complexing Agent: Gamma-cyclodextrin (CD); Molar Ratios (THY:CD): 1:1 and 2:1	Dimethylformamide (DMF)	Electrospinning	Voltage: 17 kV; Distance: 17 cm; Flow Rate: 0.5 mL/h; Temperature: 25 °C; Relative Humidity: 18%	XRD; TGA; 1H-NMR; SEM; Molecular Modeling (DFT); Release (HS GC-MS at 37, 50, 75 °C); Antibacterial Activity (*E. coli*, *S. aureus* on meat samples)	Fiber Diameter: Pure Zein 155 ± 30 nm, Zein-THY 205 ± 50 nm, Zein-THY/CD-IC (2:1) 415 ± 100 nm; Complexation Energy (Ecomp in water): −29.2 kcal/mol (1:1), −35.2 kcal/mol (2:1); Thymol Retention (TGA): 99.5% (2:1 complex) vs. 89.7% (1:1 complex) Production Rate (Yield): ~0.6 g/h (single nozzle)	Encapsulation of Thymol/gamma-CD complex (2:1 molar ratio) in zein fibers provided the highest thermal stability, controlled release, and effective antibacterial performance on meat samples	Processing: Addition of inclusion complexes (IC) increased solution viscosity and decreased conductivity, causing a significant increase in fiber diameter (from 155 nm to 415 nm) Low production throughput (~0.6 g/h) limits industrial adoption without multi-nozzle scale-up
Aytac et al., 2020 [2]	Biodegradable and antimicrobial electrospun zein fibers for food packaging (“Green” synthesis)	Polymer: Zein (from maize, Z3625) (30% *w*/*v* for pure, 37% *w*/*v* for antimicrobial); Active Cocktail: 1. Thyme oil (1% *w*/*v*), 2. Citric acid (5% *w*/*v*), 3. Nisin (0.005% *w*/*v* pure nisin)	Acetic acid (Glacial)	Electrospinning (One-step synthesis)	Flow Rate: 0.7 mL/h (Pure), 0.5 mL/h (Antimicrobial); Voltage: 26 kV (+25 kV needle, −1 kV collector); Distance: 15 cm; Needle: 0.6 mm (90° blunt end)	SEM (Morphology, ImageJ); BET (Surface Area); XRD; ATR-FTIR; Antimicrobial activity (Disk diffusion, Direct contact); Release kinetics (LC/HRMS in Water, 3% Acetic acid, 50% Ethanol)	Fiber Diameter: Zein (ZF) 140 ± 40 nm, Antimicrobial Zein (AZF) 165 ± 35 nm; Specific Surface Area (BET): 21.91 m^2^/g (AZF); Antimicrobial Reduction: ~5 log (*E. coli*, 24 h), ~5 log (*L. innocua*, 1 h); Active Loading: 2.50 mg/cm^2^; Yield: Up to 1 g/h	Zein fibers containing naturally derived antimicrobials (thyme oil, citric acid, nisin) effectively inactivated food pathogens (~5 log reduction) and represent a sustainable packaging material produced using non-toxic solvents	Analysis: Nisin release could not be reliably measured in 50% ethanol simulant due to matrix effects; Scalability: Electrospinning scalability remains a general challenge (addressed here via multi-needle, but noted as a hurdle)
Ansarifar & Moradinezhad, 2022 [14]	Encapsulation of thyme essential oil (TEO) in zein fibers for strawberry preservation	Polymer: Zein (30% *w*/*v*); Active Agent: Thyme Essential Oil (TEO) (4% *v*/*w* relative to polymer)	Glacial acetic acid	Electrospinning	Voltage: 16 kV; Distance: 15 cm; Flow Rate: 0.2 mL/h; Solvent System: Glacial acetic acid.	SEM (Morphology, 20 kV); UV-Vis (Encapsulation Efficiency at 278 nm, Release profile); Disc diffusion (MIC); Fruit Quality (Weight loss, Firmness, TSS, Anthocyanin, Color)	Fiber Diameter: Pure Zein 195.0 ± 32.1 nm, Zein/TEO 402.3 ± 26.6 nm; Encapsulation Efficiency: 75.23 ± 16.4%; Release: ~65% at 180 h; MIC: *E. coli* 4.35 ± 0.15 mg/mL, *B. cereus* 3.42 ± 0.25 mg/mL; Strawberry Weight Loss (15 days): 7.35% (Zein/TEO) vs. 12.39% (Control); Firmness (15 days): 1.34 N (Zein/TEO, highest value)	Zein/TEO fiber packaging provided controlled release of TEO, significantly reducing weight loss (by ~15%) and maintaining firmness and anthocyanin content in strawberries during cold storage	Processing: Addition of TEO decreased electrical conductivity, causing a significant increase in fiber diameter (~2-fold increase)
da Trindade et al., 2024 [15]	Electrospun polysaccharide-protein fibers for plant-based meat analogs	Zein Solution: 23 wt%; Polysaccharide Solutions: 1 wt% (Sodium Alginate, kappa-Carrageenan, HM-Pectin, or LM-Pectin); PEO Solution: 4 wt%; Blends (Zein:Polysaccharide): 80:20, 85:15, 90:10; Additive: PEO (0.3 wt% or 1 wt% added to blend)	Zein: 80% Ethanol (aqueous); Polysaccharides/PEO: Deionized Water	Electrospinning	Voltage: 18–24 kV (Optimized ~22–24 kV); Flow Rate: 1800–3000 µL/h; Distance: 12–15 cm; Needle: 1.03 mm ID; Temperature: 20–25 °C; RH: 50–60%	Rheology; SEM (Morphology); ATR-FTIR; TGA; Water Contact Angle (WCA, 25 °C)	Fiber Diameter (Zein/Carrageenan 90:10, 1% PEO): 8.86 ± 3.48 µm; WCA: Zein/PEO (97.3°), Zein/Carrageenan (90:10, 1% PEO) (65.8°), Zein/LM Pectin (15.22°); Thermal Degradation (Tonset): ~280 °C (Zein/Carrageenan)	The Zein/kappa-Carrageenan (90:10) fiber with 1% PEO offered the optimal balance of homogeneous structure, thermal stability, and surface hydrophilicity for simulating plant-based meat textures	Processing: Pure polysaccharide solutions and Alginate/PEO blends failed to form fibers (dripping); higher polysaccharide concentrations in zein blends often reduced fiber formation or homogeneity
Ghasemi et al., 2022 [16]	Encapsulation of cumin essential oil (CEO) in zein electrospun fibers for active food packaging	Polymer: Zein (27% *w*/*v* solution); Active Agent: Cumin Essential Oil (CEO) (0, 2.5, 5, 10, 20% *v/v* relative to solution)	Ethanol	Electrospinning	Polymer Concentration: 27% *w*/*v*; Voltage: 20 kV; Distance: 15 cm; Flow Rate: 0.4 mL/h; Solvent System: Ethanol (80% *v*/*v*) aqueous solution.	SEM (Morphology); AFM; XRD; DSC; FTIR; BET (Pore size); Mechanical testing (Tensile strength); Antibacterial (Disc diffusion against *S. aureus*, *E. coli*, *B. cereus*, *S. enterica*)	Fiber Diameter: Increased from 459 nm (Pure Zein) to 855 nm (20% CEO); Tensile Strength: Increased from 0.28 MPa (Pure) to 3.55 MPa (20% CEO); Pore Size (BET): Increased from 7 nm (Pure) to 13 nm (20% CEO)	Encapsulation of cumin essential oil significantly improved the mechanical strength (tensile) and thermal properties of zein fibers while imparting antibacterial activity against multiple food pathogens	Environmental conditions (RH/temperature) and collector details are not specified in the extracted text
Gomez-Caturla et al., 2022 [17]	Development of Mango Kernel Starch (MKS) based nanofibers	Polymers: Mango Kernel Starch (MKS) (0, 2, 3, 4, 5 wt%); Polyvinyl alcohol (PVA) (10, 8, 7, 6, 5 wt%) OR Polyvinylpyrrolidone (PVP) (10, 8, 7, 6, 5 wt%); Total Concentration: 10 wt%	MKS/PVA: Distilled Water; MKS/PVP: Methoxyethanol	Electrospinning	Voltage: 18–36 kV (Variable); Flow Rate: 0.05–0.75 mL/h; Distance: 13–18 cm	FESEM (Morphology); AFM (Topography, Roughness); ATR-FTIR	Fiber Diameter (MKS/PVA): 0.146 µm (thinnest)–0.315 µm; Fiber Diameter (MKS/PVP): 0.080–0.339 µm; Roughness: 80–343 nm	MKS was successfully electrospun into nanofibers when blended with synthetic polymers (PVA or PVP); for PVA blends, 3% MKS was identified as the “smooth fiber concentration threshold”	Material: At 5 wt% MKS concentration (for PVP blends), beaded fibers formed due to excessive starch content
Göksen et al., 2020 [18]	Antimicrobial zein nanofiber coatings loaded with essential oils for cheese slices	Matrix: Zein (25% *w*/*v*); Active Agents: *Laurus nobilis* essential oil (LEO) (1, 5, 10% *w*/*w*) OR *Rosmarinus officinalis* essential oil (REO) (1, 5, 10% *w*/*w*); Main Component: 1,8-cineole (69.87% in LEO, 55.80% in REO)	Glacial acetic acid/Ethanol (30:70 *v*/*v*)	Electrospinning	Voltage: 18 kV; Distance: 11 cm; Flow Rate: 0.04 mL/h; Temperature: Room temperature	SEM (ImageJ); Viscosity; Conductivity; FTIR; TGA; Encapsulation Efficiency (EE); Antimicrobial Test (*L. monocytogenes*, *S. aureus* on cheese, 4 °C, 28 days)	Fiber Diameter: Pure Zein (201.78 ± 52.24 nm), Zein/LEO 10% (118.63 ± 43.8 nm), Zein/REO 10% (127.72 ± 44.3 nm); Encapsulation Efficiency: LEO 1% (83.06%), REO 1% (81.92%); Antimicrobial Effect (Day 28): ~2 log reduction in *L. monocytogenes* and *S. aureus* vs. control	Electrospun zein nanofibers containing 1,8-cineole rich LEO and REO provided effective protection against pathogens on cheese for 28 days, offering more sustainable release compared to cast films	Material: As essential oil concentration increased (from 1% to 10%), encapsulation efficiency showed a statistically significant decrease (dropping from ~83% to ~74%)
Li et al., 2023 [19]	Electrospinnability of commercial OS starches (waxy maize origin) blended with Pullulan	Polymers: OS starches (PGU, PG2000, HC100); Pullulan (PUL); Blends: PGU-PUL (e.g., PGU 12–20% *w/v* with PUL 12% *w*/*v*; PGU 15% with PUL 8–13%)	Deionized water (aqueous dispersions)	Electrospinning	General Range: Feed 0.1–0.4 mL/h, Distance 5–10 cm, Voltage 5–15 kV; Optimized (30% PGU): Voltage 24 kV, Distance 9 cm, Flow rate 0.2 mL/h; Needle: 22 G blunt; Dope Heating: Boiling water bath (1 h), cooled to ~20 °C	SEM (5 keV, ImageJ); Rheology (ARES); Surface tension (Pendant drop); Conductivity	Fiber Diameter: 150 ± 34 nm → 250 ± 41 nm (PGU 12% → 20%, PUL fixed); 147 ± 26 nm → 209 ± 57 nm (PGU fixed, PUL 8% → 13%); Viscosity: 1.52–9.76 Pa·s (Electrospinnable range); Lowest Viscosity Required: 1.52 Pa·s (PGU 12%)	Additions of a relatively small amount of Pullulan to food-grade starch allow for the creation of continuous, smooth, and bead-free nanofibers via a “green” aqueous process	Analysis: Further studies are suggested to focus on the physicochemical properties of the starch-based nanofibers and their utilization
Shahbazi et al., 2024 [20]	Essential oil loaded zein nanofibers for minced meat packaging	Polymer: Zein (26 g/100 mL); Active Agents: *Foeniculum vulgare* essential oil (FVO) (1 mL/100 mL), *Carum carvi* essential oil (CCO) (1 mL/100 mL); Combination: Zein + FVO + CCO	Ethanol: Distilled water (80:20, *v*/*v*)	Electrospinning	Voltage: 18 kV; Flow Rate: 0.80 mL/h; Distance: 22 cm; Rotation Speed: 0.50 × g (Collector); Temperature: 25 ± 1 °C	SEM (Morphology, ImageJ); FTIR; Mechanical Test (TS, EAB); Water Vapor Permeability (WVP); Antimicrobial (Minced meat, *S. aureus*, *L. monocytogenes*, *Y. enterocolitica*, 10 days, 4 °C)	Thickness: 0.20–0.22 mm; Tensile Strength (TS): 6.01–9.22 MPa; Elongation at Break (EAB): 5.57–10.78%; WVP: 1.05–2.72 × 10^−14^ kg m/m^2^ s Pa; Bacterial Reduction (Day 10): Combined fibers reduced *S. aureus* to <1.00 log CFU/g (from 5 log start)	Zein nanofibers loaded with FVO and CCO significantly delayed bacterial growth in minced meat during cold storage, showing potential for antimicrobial food packaging	NR—No specific limitations or missing experiments were explicitly highlighted in the snippet
Vitoria et al., 2025 [21]	Active bilayer food packaging (Starch film + Zein fibers)	Layer 1 (Film): Sweet potato starch (4.0% *w*/*v*); Layer 2 (Fibers): Zein (30% *w*/*v*) loaded with Thyme Essential Oil (TEO) (60% *v*/*w*)	Starch: Water; Zein: Ethanol	Hybrid (Electrospinning on Cast Film)	Zein Concentration: 30% *w*/*v*; Voltage: NR	SEM; FTIR; TGA; Water vapor permeability (WVP); Mechanical strength; Antioxidant activity	Loading Capacity: 33.2%; Encapsulation Efficiency: 91.0%; Thickness: 0.194 mm; Active Component: p-cymene (36.4% in TEO)	The bilayer design integrates the structural properties of starch film with the bioactive functions (antioxidant, controlled release) of zein fibers, preserving biodegradability	Gap: Real food storage performance and long-term stability were not tested in this study (suggested for future work)
Wu et al., 2023 [8]	Context (zein-only) Effect of pH on the structure, rheology, and electrospinning of maize zein	Polymer: Maize Zein (10–32.5% *w*/*v*); pH Adjustment: pH 4, 5, 6, 7, 8 (adjusted with 0.01 mol/L HCl and NaOH)	80% (*v*/*v*) Ethanol aqueous solution	Electrospinning	Voltage: 15 kV; Flow Rate: 6 mL/h; Distance: 15 cm; Needle: 21 G (0.5 mm ID); Temp: 25 ± 2 °C; RH: 50 ± 5%	Rheology (0.01–1000 s^−1^); Conductivity; SEM (ImageJ); FT-IR; XRD; Water Contact Angle (2 µL); AFM (Roughness)	Critical Entanglement Conc. (Ce): 17.6% (pH 4), 20.1% (pH 5/6), 17.1% (pH 7), 19.5% (pH 8); Fiber Diameter (30% *w*/*v*): 321 nm (pH 5, Min), 0.476 µm (pH 8, Max); Secondary Structure (pH 6): β-sheet 45.47% (Highest), α-helix 18.58% (Lowest)	Zein solutions can be stabilized to form electrospun fibers at a variety of pH levels (4–8); while pH had less effect on spinnability, it significantly influenced the chemical/physical properties (roughness, secondary structure)	Processing: At pH 6 (near isoelectric point), the viscosity increase was caused by protein aggregation rather than molecular entanglement, preventing perfect bead-free fiber formation at the inflection point

Note: The table summarizes material systems, solvent classes, electrospinning configurations, tested functionalities, and explicitly reported limitations. Context-only studies (starch-only, zein-only, or multilayer reference systems) are included solely for mechanistic benchmarking and are excluded from all quantitative synthesis.

**Table 2:** The Solvent–Morphology Trade-off.

Solvent System Category	Studies Analyzed (n)	Avg. Fiber Diameter (nm)	Fiber Range (nm)	“Green” Status
Acetic Acid/Acidic	3	228 ± 152	118–402	Caution (Corrosive/Smell)
Ethanol/Aqueous	6	341 ± 132	200–539	High (Food Grade)
Toxic/Organic (e.g., DMF)	1	415	415	Low (Restricted)

Note: Data are derived exclusively from the evaluated core corpus of electrospun starch–zein composite studies summarized in Table 1 (2014–2025); context-only systems were excluded from all quantitative averages. Note: The high standard deviation (±152 nm) in the Acidic/Acetate system reflects the aggregation of divergent solvent protocols. While acetic acid promotes uniform protein unfolding yielding fine fibers [18], formic acid induces starch depolymerization and formylation [10], creating a broader distribution of fiber diameters due to variable solution viscosity.

**Table 3:** Process-Structure-Feature Correlations in Electrospun Zein Systems.

Parameter Change	Primary Feature Affected	Quantitative Observation/Trend	Source
↑ Viscosity (TEO Loading)	Fiber Diameter	Increase from 195 nm to 402 nm	[14]
↑ Conductivity (Carvacrol)	Fiber Diameter	Decrease from 604 nm to 539 nm	[12]
pH Shift (4 to 6)	Protein Structure	β-sheet increase (36% to 45%); R_q_ decrease	[15]
Electrospinning vs. Casting	Wettability	WCA increase from <60° to >100°	[15]
Bioactive Loading	Crystallinity	Complete amorphization (loss of XRD peaks)	[11,14]

Note: The table summarizes material systems, solvent classes, electrospinning configurations, tested functionalities, and explicitly reported limitations. Context-only studies (starch-only, zein-only, or multilayer reference systems) are included solely for mechanistic benchmarking and are excluded from all quantitative synthesis. Arrows indicate the direction of change in the parameter (↑ increase).

**Table 4:** Comparative Synthesis of Structural Architectures in Zein-Based Systems.

Architecture	Polymer Composition	Active Agent Strategy	Functional Advantage	Reference
Monolithic	Zein + TEO (Emulsion)	Homogeneous dispersion	Reducing weight loss in fruit (Strawberry)	[14]
Monolithic	Zein + Polysaccharides	Structural blend (No active)	Textural modification for meat analogs	[15]
Core–Shell	Shell: Zein/Core: PEO	Ferulic Acid (in Core)	Zero-order sustained release profile	[29]
Core–Shell	Shell: PCL/Core: Starch	None (Scaffold focus)	Mechanical support for starch fiber formation	[28]
Bilayer	Starch Film + Zein Fiber	TEO (in Fiber layer)	Combined gas barrier and antioxidant activity	[21]
Janus	PCL side/CA side	Lavender Oil + AgNPs	Synergistic dual-agent delivery	[31]
Janus	PVP side/EC side	Ketoprofen + Dye	Biphasic (Fast + Slow) release kinetics	[30]

**Table 5:** The ‘Functionality Gap’ Matrix for the Evaluated Electrospun Starch–Zein Corpus (2014–2025).

Research Metric	Frequency in Core Corpus	The “Why” (Diagnosis)
Dry Morphology (SEM)	~100%	Easy to perform; produces publication-ready images.
Active Release (Buffer)	~75%	Standardized lab protocols exist; low complexity.
Wet-State Mechanics	<10%	Difficult to handle plasticized/weak fibers; requires specialized environmental chambers.
Solvent Residue Analysis	<5%	Often ignored in academic labs; critical for FDA/EFSA compliance.
Real Food Interaction	~25%	Requires complex biological handling (e.g., meat/fish) vs. simple disk diffusion.

**Table 6:** The Impact of Bioactive Cargo on Fiber Morphology: A Study in Contradictions (2014–2025).

Reference	Active Agent	Concentration	Effect on Diameter	Proposed Mechanism (Author’s Claim)
[14]	Thyme Essential Oil	4% *v*/*w*	Increase (↑ 2-Fold) (195 nm → 402 nm)	Reduced conductivity prevents jet stretching
[16]	Cumin Essential Oil	20% *v*/*v*	Increase (↑ 85%) (459 nm → 855 nm)	Increased viscosity dominates the process
[12]	Carvacrol	10% *w*/*w*	Decrease (↓ 10%) (604 nm → 539 nm)	Plasticization effect reduces solution viscosity
[18]	Laurus nobilis Oil	10% *w*/*w*	Decrease (↓ 40%) (201 nm → 118 nm)	Conductivity changes favored stretching
[20]	Fennel & Caraway Essential Oils	5–20% (*w*/*v*)	Increase (272 nm → 614 nm)	Increased solution viscosity and total solids content led to thicker fiber formation

Note: Arrows indicate the direction of change in the parameter (↑ increase; ↓ decrease).

**Table 7:** Mechanical Paradox: Reinforcement vs. Plasticization in Zein Nanocomposites.

Study	Matrix System	Active Agent	Tensile Strength (MPa)	Outcome
[16]	Pure Zein	None	0.28 MPa	Reference (Weak)
[16]	Zein + CEO	Cumin Oil (20%)	3.55 MPa	12× Reinforcement (Strong intermolecular interaction)
[16]	Zein + Oils	Fennel + Caraway	6.01–9.22 MPa	High mechanical integrity despite oil load
General Trend	Starch/Zein	Various		>80% of studies fail to report this metric

**Table 8:** The Evolutionary Trajectory of Electrospun Food Packaging (2017–2025).

Development Stage	Focus Era	Key Characteristic	Representative Approach	Limitation
Gen 1: Morphology	2014–2018	Fiber Diameter Optimization	Solvent selection (e.g., Acetic acid vs. Ethanol) [26]	Non-functional; pure structural focus.
Gen 2: Encapsulation	2018–2022	Passive Loading of Actives	Loading Essential Oils, Vitamins (B9), Phenolics [6,13,41]	“Burst release” or uncontrolled diffusion.
Gen 3: Architecture	2020–2023	Multilayer & Hybrid Systems	Sequential electrospinning (Layer-by-Layer) [44,45]	Complex manufacturing; delamination risks.
Gen 4: Intelligence	2023–Future	Stimuli-Responsive Systems	Air-assisted scaling; Responsive release [19]	Current research frontier.

**Table 9:** Techno-Economic Assessment of Scale-Up Technologies for Zein/Starch Systems.

	Representative Study	Throughput (g/h)	Product Uniformity	Operational Challenge	Cost & Safety Risk	Readiness Level (TRL)
Single Needle (Lab Standard)	Baseline	~0.12	High (165 ± 35 nm)	Low	Low Cost/High Safety	Academic Only
Multi-Needle (20-Emitter)	[2]	~1.00	High (160 ± 40 nm)	High (Field Interference & Clogging)	Medium Cost/High Safety	Pilot Scale
Needleless (Free Surface)	[32]	>10.0	Low/Medium (Variable Dia.)	Low (No clogging)	High Cost/Solvent Vapor Risk	Mass Production

Note: Calculated based on typical polymer concentrations (20–30%).

## Data Availability

No new data were created or analyzed in this study. Data sharing is not applicable to this article.

## Supplementary Materials

The following supporting information can be downloaded at https://www.mdpi.com/article/10.3390/polym18070823/s1. Figure S1: PRISMA flow diagram detailing the literature search, screening, and selection process used to identify the core corpus of 82 starch–zein electrospinning studies (2014–2025).

## Abbreviations

The following abbreviations are used in this manuscript:

EHDP	Electrohydrodynamic Processing
SEM	Scanning Electron Microscopy
XRD	X-ray Diffraction
FTIR	Fourier Transform Infrared Spectroscopy
RH	Relative Humidity
GRAS	Generally Recognized As Safe
WVP	Water Vapor Permeability
CA	Citric Acid
W/O/W	Water-in-Oil-in-Water
TRL	Technology Readiness Level
CEO	Cumin Essential Oil
TEO	Thyme Essential Oil
