# Supplementary material for: Navigating the Bio-Composite Landscape: A Strategic Reconstruction of Electrospun Starch–Zein Nanofibers
Source: Polymers (Basel). 2026 Mar 27;18(7):823. doi: 10.3390/polym18070823 (PMC13074667; doi:10.3390/polym18070823)
Supplement: Supplementary file 1 [file polymers-18-00823-s001.zip › polymers-4123690-supplementary.pdf]

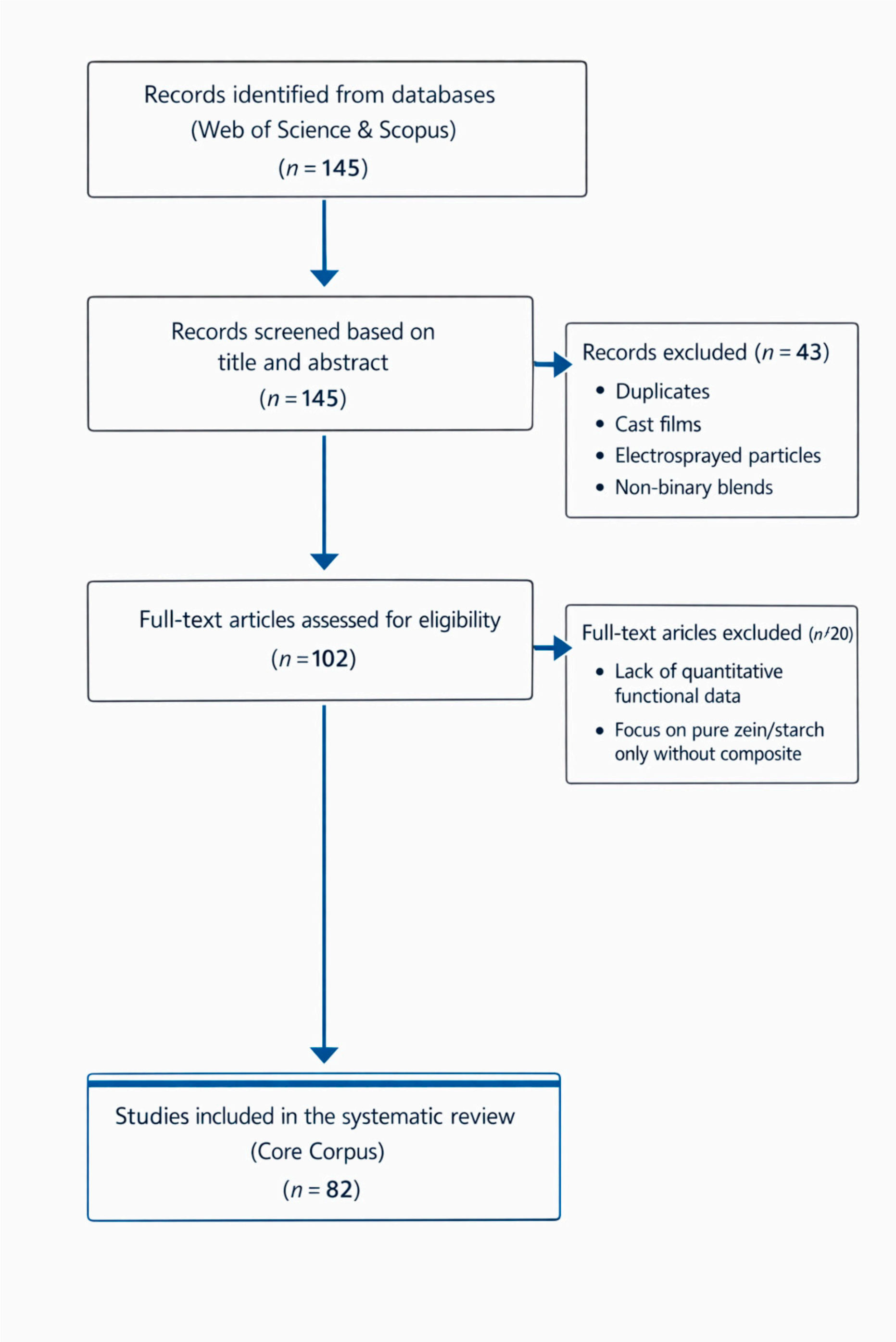

Figure S1. PRISMA flow diagram detailing the literature search, screening, and selection process used to identify the core corpus of 82 starch–zein electrospinning studies (2014–2025)
